# How unique is the low oxygen response? An analysis of the anaerobic response during germination and comparison with abiotic stress in rice and Arabidopsis

**DOI:** 10.3389/fpls.2013.00349

**Published:** 2013-10-01

**Authors:** Reena Narsai, James Whelan

**Affiliations:** ^1^Plant Energy Biology, Centre for Computational Systems Biology, University of Western AustraliaPerth, WA, Australia; ^2^Department of Botany, School of Life Science, La Trobe UniversityMelbourne, VIC, Australia

**Keywords:** low oxygen, transcriptomes, rice, Arabidopsis, microarrays

## Abstract

Plants face a variety of environmental stresses and have evolved molecular mechanisms to survive these challenges. One of these stresses is low oxygen conditions, which can occur under flooding conditions. Rice (*Oryza sativa*) is somewhat unique for its ability to tolerate and even germinate under low to no oxygen conditions. In this study, we examined global transcriptomic responses over the course of germination and in response to low oxygen and other abiotic stress in rice and Arabidopsis (*Arabidopsis thaliana*). Over 150 microarray datasets were analyzed in parallel to determine just how unique the low oxygen response is in rice. Comparison of aerobic germination in rice and Arabidopsis, with anaerobic germination in rice revealed conserved transcriptomic responses that are not only conserved across both species but also occur in the absence of oxygen in rice. Thus, these genes may represent functions necessary for the developmental progression of germination, whether or not oxygen is present in rice. Analysis of genes that responded differently in rice compared to Arabidopsis revealed responses specific to anaerobic germination in rice, including the down-regulation of genes encoding redox functions and up-regulation of receptor kinases. Comparison of a range of hypoxia/anoxia studies within and across Arabidopsis and rice revealed both conserved and species specific changes in gene expression (e.g., Arabidopsis specific up-regulation of WRKYs and rice specific down-regulation of heme), unveiling unique transcriptomic signatures of the low oxygen response. Lastly, a comparison of the low oxygen response with cold, salt, drought and heat stress revealed some similarity with the response to heat stress in Arabidopsis, which was not seen in rice. Comparison of these heat-responsive, abiotic stress marker genes in Arabidopsis with their rice orthologs revealed that while low oxygen may be perceived as an abiotic stress in Arabidopsis, this is not the case in rice.

## Introduction

Plants can experience low oxygen conditions at various times during the plant life cycle, from early development (such as germination) due to diffusional resistance to oxygen resulting from anatomical restrictions, or more commonly due to partial or complete submergence under flooding conditions. Several species of plants, including some varieties of rice (*Oryza sativa*), have the ability to germinate, grow and survive under oxygen limiting conditions. However, most other plant species including Arabidopsis (*Arabidopsis thaliana*) are highly intolerant of low oxygen conditions. Interestingly, despite the different levels of low oxygen tolerance across different plant species, common responses to oxygen limitation are also often observed (Mustroph et al., 2010) including the shift to fermentation from mitochondrial respiration, as well as other multi-level molecular changes that help limit energy demanding processes in an attempt to prolong survival (Bailey-Serres and Voesenek, 2008; Magneschi and Perata, 2008; Mustroph et al., 2010). By far the most well-characterized response to low oxygen is the activation of fermentation involving an increase in alcohol dehydrogenase abundance (Sachs et al., 1980). Additionally, an increase in glycolytic flux, lactate dehydrogenase and pyruvate dehydrogenase are also seen under low oxygen stress in plants (Rivoal et al., 1991; Gibbs et al., 2000). Sucrose degradation is also modified under low oxygen, whereby sucrose is metabolized by sucrose synthase in an attempt to conserve ATP (Ricard et al., 1991; Magneschi and Perata, 2008). Similarly, a switch to pyrophosphate (PPi) linked enzymes is also made as PPi is adopted over ATP as high-energy donor molecules under low oxygen conditions (Huang et al., 2008).

In both plants and animals, nitrite is also reduced to form nitric oxide (NO) under low oxygen conditions (Sturms et al., 2011). Studies in plants have implied a role for cytochrome oxidase, cytochrome bc1, and non-symbiotic hemoglobins in this reaction (Igamberdiev and Hill, 2009; Igamberdiev et al., 2010; Igamberdiev and Kleczkowski, 2011; Sturms et al., 2011). While the role of non-symbiotic hemoglobins has not been fully elucidated, there is increasing evidence for a function in NO scavenging, with a recent study even confirming significantly faster rates of hemoglobin activity under low oxygen conditions in rice plants compared to animals (Igamberdiev and Hill, 2009; Sturms et al., 2011). Thus, these changes in NO metabolism and ATP synthesis present an alternative method for supporting the redox and energy balance under low oxygen conditions in rice.

Given this general response of limiting energy demanding processes under low oxygen conditions, it is unique that rice has the ability to germinate under the complete absence of oxygen. Germination under aerobic conditions has been well-characterized at the transcript and protein levels in rice (Howell et al., 2006, 2009) and Arabidopsis (Gallardo et al., 2002; Nakabayashi et al., 2005; Narsai et al., 2011a). As a high energy demanding process, germination is often characterized by the significant up-regulation of mitochondrial respiratory chain and glycolysis components (Howell et al., 2007, 2009) in order to produce the energy required for development. While it may be expected that germination occurs more slowly under low oxygen conditions, in fact, under anaerobic germination in rice, morphological changes such as accelerated shoot elongation is observed and aerenchyma is developed to efficiently deliver oxygen from the shoot to the submerged organs (Magneschi and Perata, 2008). In addition to these morphological adaptations, the typical increases in alcohol dehydrogenase, pyruvate decarboxylase and lactate dehydrogenase also occurs (Magneschi and Perata, 2008). Thus, significant metabolic and molecular re-programming must occur to generate the energy required for both germination and rapid shoot elongation under low oxygen in rice.

It has previously been shown that a significant amount of transcriptomic re-programming occurs under low oxygen conditions in various species (Mustroph et al., 2010; Narsai et al., 2011b). Thus, it is not surprising that transcription factors, such as ethylene response transcription factors (ERFs) have been shown to have a key role in the response to low oxygen in plants. The Sub1A locus encoding an ERF was the first gene shown to confer submergence tolerance in certain rice cultivars by altering the expression of specific low oxygen responsive genes including the alcohol dehydrogenase I encoding genes (Xu et al., 2006). Similarly, it was shown that group VII ERFs, containing a conserved N-terminal motif have an important role in oxygen sensing and mediating the low oxygen response in Arabidopsis (Gibbs et al., 2011). It was shown that the N-end rule pathway of targeted proteolysis acts as an oxygen sensor, where plants lacking the constituents of this pathway were more tolerant to hypoxia (Gibbs et al., 2011). This tolerance was also shown to occur as a result of an increase in the expression of core hypoxia response genes (Gibbs et al., 2011). Considering these findings, it is clear that transcriptional reprogramming is a core component of the hypoxia response in plants, with many studies observing substantial transcriptomic changes in response to low oxygen in rice (Lasanthi-Kudahettige et al., 2007; Magneschi and Perata, 2008; Narsai et al., 2009), Arabidopsis (Loreti et al., 2005; Branco-Price et al., 2008; Christianson et al., 2009, 2010; Banti et al., 2010) and several other plant species (Mustroph et al., 2010; Banti et al., 2013).

While significant transcriptomic changes do occur under low oxygen conditions, it is important to note that many of these are not exclusively due to low oxygen stress. In fact, transcription factors can often regulate responses that confer tolerances to multiple abiotic stresses. For example, WRKY transcription factors, such as WRKY18 and WRKY60 have been shown to have a role under both salt and osmotic stress (Chen et al., 2010). Similarly, it has been shown that while the Sub1A locus confers submergence tolerance in rice, it also has a role in drought tolerance (Fukao et al., 2011). Likewise, it has been shown that the heat shock factor, HsfA2 also, has a role in enhancing anoxia tolerance in Arabidopsis (Banti et al., 2010). Notably, this gene was identified when the transcriptomic responses to heat and anoxia were compared to identify common responses (Banti et al., 2008, 2010).

In the present study, we aimed to gain insight into the specificity of the transcriptomic response to low oxygen in rice. Over 150 microarrays from various studies were analyzed in parallel, comparing the transcriptomic responses to low oxygen conditions within and across Arabidopsis and rice. Firstly, core transcriptomic changes during germination were examined under aerobic and anaerobic conditions in rice (Howell et al., 2009; Narsai et al., 2009) as well as under aerobic germination in Arabidopsis (Narsai et al., 2011a). Therefore, developmental responses could be identified and separated from the specific anaerobic responses during germination. Also, core oxygen dependent developmental responses over the course of anaerobic germination were identified. Furthermore, multiple hypoxia/anoxia datasets were combined for both rice and Arabidopsis to reveal common and distinct functional categories affected under low oxygen conditions between both species. Lastly, the expression of Arabidopsis abiotic stress marker genes were examined in response to hypoxia and cold, drought, salt and heat, revealing common responses between low oxygen and other abiotic stresses. The results presented here reveal unique responses to low oxygen conditions over germination compared to other abiotic stresses.

## Results

### Oxygen independent transcriptomic changes during rice germination

**Figure 1:**
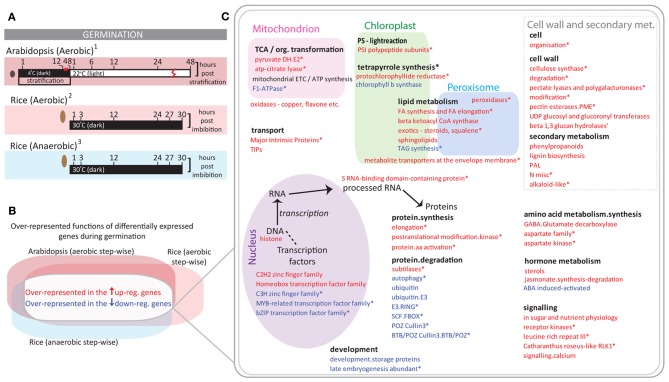
**Oxygen independent transcriptomic changes during rice germination. (A)** Step-wise comparisons were carried out over the course of aerobic germination in Arabidopsis (^1^Narsai et al., 2011a) aerobic germination in rice (^2^Howell et al., 2009) and anaerobic germination in rice (^3^Narsai et al., 2009). **(B)** The differentially expressed genes (>2-fold, *p* < 0.05, PPDE > 0.96) were analyzed for common over-representation of functions in the up/down-regulated gene-sets. **(C)** Functional categories seen to be commonly up/down-regulated across all three germination studies are shown. Red font indicates that genes encoding these function(s) are over-represented in the up-regulated gene-sets and blue indicates over-representation of functions in the down-regulated gene-sets. Categories responsive at the same time in both aerobic and anaerobic germination in rice are denoted with an asterisk.

In order to identify common changes in the transcriptomes of rice and Arabidopsis, we compared three germination studies; one from Arabidopsis (Narsai et al., 2011a), as well as aerobic germination (Howell et al., 2009) and anaerobic germination in rice (Narsai et al., 2009) (Supplementary Tables 1, 2 and Supplementary Figure 1). As these were all time course studies, step-wise differential expression analysis was carried out comparing each time point with the previous time point, only genes significantly differentially expressed by two or more fold were included (*p* < 0.05, PPDE > 0.96) (Figure 1A). These were then analyzed for over-representation of functional categories using the Pageman tool (Usadel et al., 2006) and the over-represented functional categories are shown for each comparison in Supplementary Table 1. By comparing the over-represented functional categories in each of these studies, it was possible to identify functions that showed a common response across all three germination studies (Figures 1B,C).

Common regulation of genes encoding several different functional categories was seen across these studies including the conserved down-regulation of transcripts encoding protein degradation functions, abscisic acid (ABA) responsive proteins, storage proteins, and late embryogenesis abundance proteins (Figure 1C). For some of these functions, it was seen that this regulation occurred at the same time in both aerobic and anaerobic germination in rice, denoted with an asterisk (Figure 1C). For example the up-regulation of protein synthesis functions such as protein elongation occurred between 3 and 12 h during rice germination (denoted with asterisk; Figure 1C; Supplementary Table 3). Similarly, the down-regulation of protein degradation functions also occurred between 3 and 12 h during rice germination (denoted with asterisk; Figure 1C; Supplementary Table 3). Note that the down-regulation of these functions is also well-known in other plant species during germination (Catusse et al., 2008; Sreenivasulu et al., 2008). However, while the same up-regulation and down-regulation patterns were seen for these during germination in Arabidopsis as well, these occurred between 12 and 48 h of cold, dark stratification in Arabidopsis (Narsai et al., 2011a). Thus, the exact timing and conditions must be considered in these comparisons.

It is important to note that a number of light responsive genes were differentially expressed during Arabidopsis germination, whilst the rice orthologs to these did not show this expression pattern during rice germination, given that the rice germination studies were carried out in the dark. Nevertheless, some photosystem I polypeptide subunits were seen to be induced during germination in both rice and Arabidopsis (Figures 1A,C). However, closer examination reveals that this induction occurs between 3 and 12 h in rice, whilst this largely occurs after 12 h into the light (after 48 h stratification) in Arabidopsis. Thus, any common induction of light-responsive genes is likely to be due to a small amount of light exposure on the rice seeds, possibly occurring during the sample collection process. Whereas, if rice germination had occurred under light conditions, this induction may have taken place later, upon, and after radicle emergence (as is seen in Arabidopsis). Interestingly, despite the fact that rice is a starch seed and Arabidopsis is an oil seed, a conserved up-regulation of lipid metabolism functions was also seen, for example, genes encoding fatty acid (FA) synthesis and elongation functions, sphingolipid metabolism and peroxidases were up-regulated in both Arabidopsis and rice (aerobic and anaerobic germination) (Figure 1C). Thus, finding these functions to be responsive in the same manner under anaerobic conditions (and in the dark) in rice indicates that irrespective of the presence of oxygen, these represent conserved transcriptomic changes that may be necessary for germination progression.

### Identifying transcriptomic changes unique to anaerobic germination

**Figure 2:**
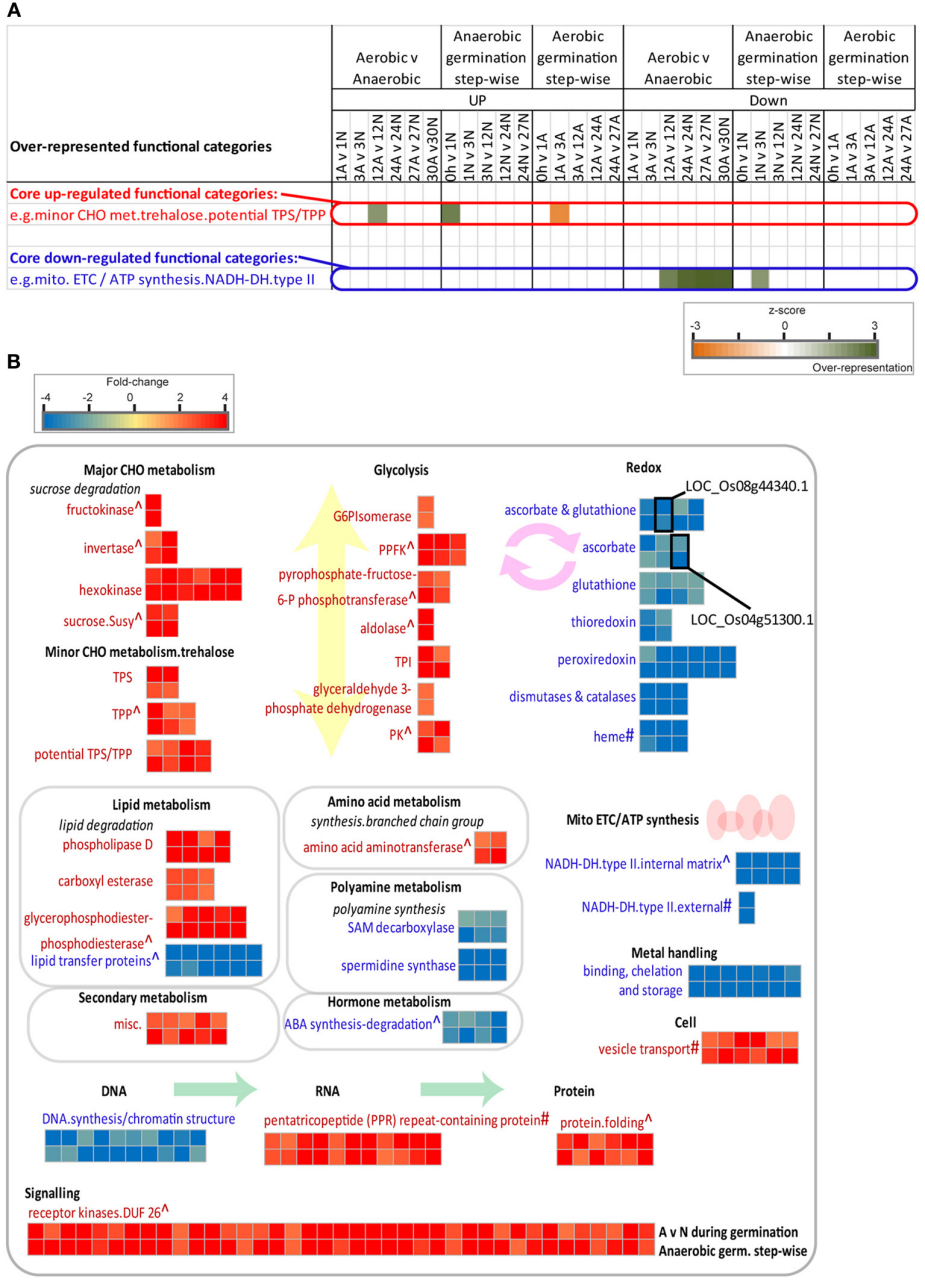
**Transcriptomic changes specific to anaerobic germination in rice. (A)** Core up/down-regulated genes exclusive to anaerobic germination were identified by isolating genes encoding functions that were over-represented in comparison to aerobic germination (Aerobic v Anaerobic), as well as over the course of anaerobic germination (step-wise), whilst not being over-represented over the course of aerobic germination (step-wise). **(B)** Differential expression of genes encoding core over-represented functions unique to anaerobic germination only [as defined in **(A)**]. Significant fold-changes (>2-fold, *p* < 0.05, PPDE > 0.96) are shown as a heatmap for genes both in comparison to aerobic germination (A v N—top rows) as well as over the course of anaerobic germination (step-wise—bottom rows). Categories showing similar responses during Arabidopsis germination are indicated with ^∧^ and categories containing genes that showed transient responses in Arabidopsis e.g., up then down or down then up, are denoted with ^#^.

After comparing the over-represented functional categories in each of the germination datasets, it also became apparent that certain functional categories were only over-represented in each of the individual germination studies. Specifically, we were interested to see which functions may be responsive under anaerobic germination only. To do this, another dataset was included for comparison which encompassed a comparison of aerobic vs. anaerobic germination (Supplementary Table 4), as well as each of the step-wise germination comparisons (Figure 2A). These combined analyses revealed functional categories that were over-represented not only compared to aerobic germination, but also over the course of anaerobic germination, showing core up-regulated and down-regulated functions specific to anaerobic germination (Figure 2A) (Supplementary Table 5). Fold-changes compared to aerobic germination, as well as over the course of anaerobic germination are shown (Figure 2B). In this way, it was revealed that there are a number of unique responses observed only during anaerobic germination in rice (Figure 2B).

For example, genes encoding redox functions showed significant down-regulation, not only over the course of anaerobic germination but also compared to aerobic germination indicating that the suppression of these occurs specifically during anaerobic germination (Figure 2B). Notably, when these categories were compared with those seen during Arabidopsis germination (Narsai et al., 2011a), it was seen that while redox functions were down-regulated in rice; several orthologous Arabidopsis genes even showed up-regulation during germination. This was particularly notable for the ascorbate glutathione cycle functions, for example while the rice gene encoding a monodehydroascorbate reductase (LOC_Os08g44340.1) was down-regulated nearly 9-fold during anaerobic germination, its Arabidopsis ortholog (At3g09940) was up-regulated 30-fold (Figure 2B). Similarly, a rice gene encoding an L-ascorbate peroxidase (LOC_Os04g51300.1) was down-regulated over 5-fold under anaerobic germination, while its Arabidopsis ortholog (At4g09010) was up-regulated 32-fold (Figure 2B). In contrast, while genes encoding various major CHO metabolism and glycolysis functions showed unique up-regulation under anaerobic germination (compared to aerobic germination) in rice, these functions were also up-regulated during germination in Arabidopsis (denoted with ^∧^ in Figure 2B). The up-regulation of major CHO metabolism and glycolysis functions specifically during anaerobic rice germination was not surprising as it a well-known response to anaerobic conditions in rice (Magneschi and Perata, 2008). Similarly, the up-regulation of protein folding functions as well as receptor kinases was also seen in Arabidopsis, but was unique to anaerobic germination in rice (Figure 2B). Interestingly, transcripts encoding pentatricopeptide repeat (PPR) containing proteins, which are involved in organelle RNA processing functions were seen to be specifically up-regulated during anaerobic germination in rice (Figure 2B). This was particularly interesting given that many PPR genes are known to be essential in Arabidopsis, where knocking out these genes often results in embryo lethality (Tzafrir et al., 2003; Khrouchtchova et al., 2012). Furthermore, it has been shown that these are transiently expressed over the course of germination in Arabidopsis, whereby a strong increase in expression is seen in the early hours after germinating seeds are transferred into light, and this expression decreases substantially by 6 h into the light (Narsai et al., 2011a) (# indicates both up- and down-regulation of genes encoding these functions in Arabidopsis; Figure 2B). Thus, the specific expression of these genes during anaerobic germination in rice may be indicative of unique RNA processing demands necessary under anaerobic conditions.

### How conserved is the low oxygen response?

**Figure 3:**
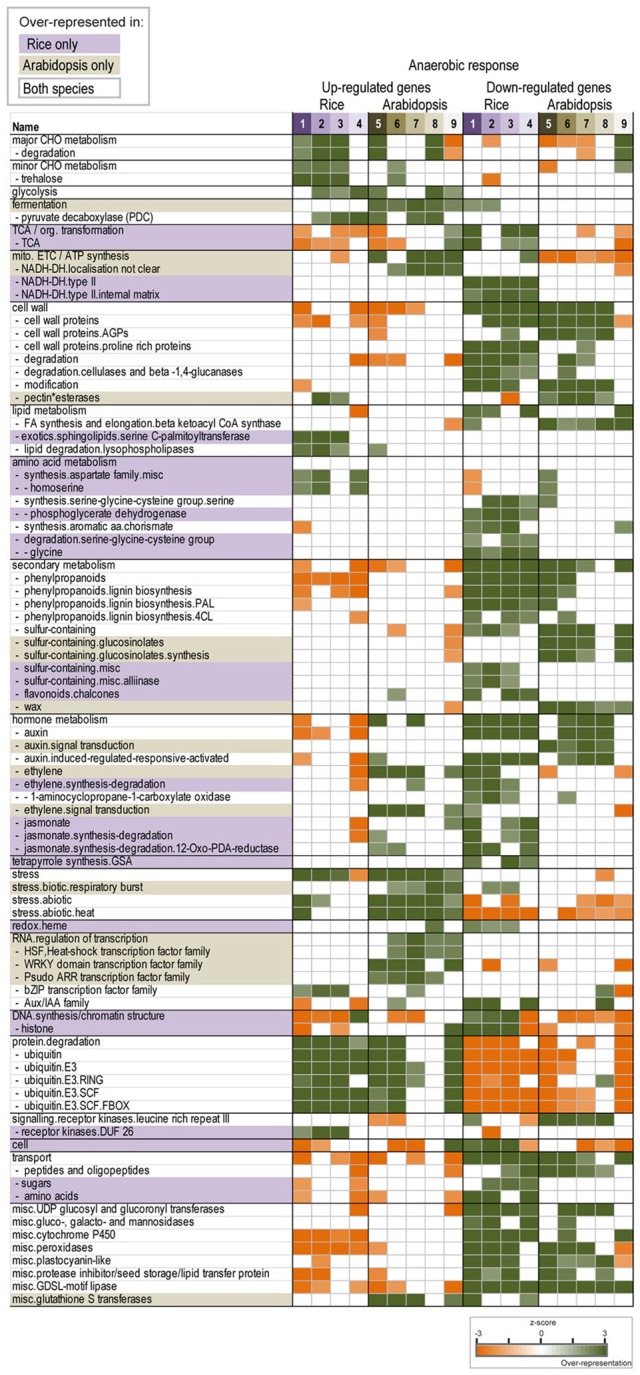
**Defining the core low oxygen response**. Pageman over-representation analysis was carried out for the significantly differentially expressed genesets (>2-fold, *p* < 0.05, PPDE > 0.96) in response to low oxygen in rice and Arabidopsis. The gene sets used included four different comparisons in rice and five different comparisons in Arabidopsis of control vs. anaerobic treatment (details in Table 1). Only the functional categories over-represented in rice only, Arabidopsis only or both species are shown (*z*-scores indicate over/under-representation, indicated by the green/yellow colors, respectively).

It has been shown that there is cross kingdom conservation of specific responses to anaerobic conditions, such as the up-regulation of the fermentative pathway (Mustroph et al., 2010). Further evidence for this was shown in Figure 2B, whereby, despite some rice genes showing specific expression not seen during aerobic germination, these expression patterns were seen for some genes during (aerobic) Arabidopsis germination. Thus, in order to examine how conserved the low oxygen response is, we compiled data from multiple studies and comparisons and analyzed the transcriptomic responses to low oxygen within and between Arabidopsis and rice (studies outlined in Table 1; data shown in Supplementary Tables 4, 6). For the rice analysis; four sets of comparisons looking at the anoxia response were used from three studies, whilst five sets of comparisons of the hypoxia/anoxia response were used from five studies in Arabidopsis (references listed in Table 1). Over-representation analysis of functional categories within the differentially expressed datasets were compared and matched, and functional categories over-represented in one or both species were identified (Figure 3). In this way, it was possible to see the expected up-regulation of genes encoding pyruvate decarboxylase (involved in glycolysis) across multiple studies within and across both Arabidopsis and rice (Figure 3). A closer look at some other components of glycolysis revealed differences in the magnitude of responses between Arabidopsis and rice, for example, the pyruvate dikinase encoding gene (LOC_Os03g31750.1), was up-regulated 117-fold during anaerobic germination, and over 360-fold in the anaerobic coleoptile; (Lasanthi-Kudahettige et al., 2007), while the Arabidopsis ortholog (At4g15530) only shows between a 2- and 16-fold induction in response to low oxygen in Arabidopsis (Supplementary Table 2). In addition to these, a conserved up-regulation of protein ubiquitination functions was also identified in both species (Figure 3). In contrast, cell wall and secondary metabolism functions were seen to be over-represented in one or more down-regulated gene sets in both species (Figure 3). Note that several of these trends were confirmed both in the individual studies that presented these microarray data (Table 1) and in the cross-kingdom analysis of responses to anoxia (Mustroph et al., 2010).

**Table 1:**
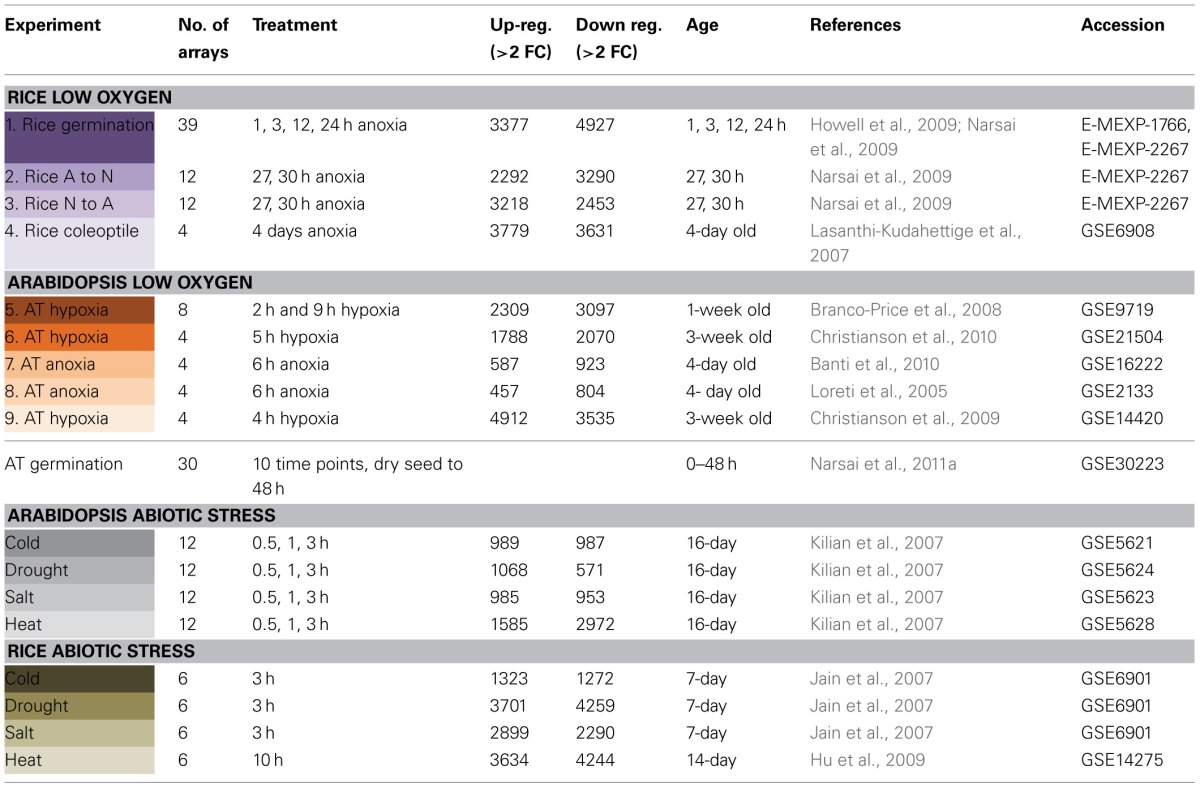
**Microarray data used for analysis**.

*For rice and Arabidopsis, various datasets examining the hypoxia/anoxia responses as well as abiotic stress responses were analyzed in parallel. The number of arrays involved in each of the comparisons/experiments, the treatments involved and the number of significantly differentially expressed genes (>2-fold) after false discovery rate correction (p < 0.05, PPDE > 0.96) are shown (up/down-regulated). The age of the plants, publications referring to these arrays and array accession numbers are shown*.

Thus, while there was some conservation in the response to low oxygen within and across Arabidopsis and rice, we also identified functional categories that appeared only to be specifically over-represented in only one species. These were highlighted as species specific when over-representation was seen in three out of four of the rice comparisons, or four out of five of the Arabidopsis comparisons (highlighted in Figure 3). For example, genes encoding NADPH type II dehydrogenases, phosphoglycerate dehydrogenase, glycine degradation (amino acid metabolism) and heme (redox) functions were seen to be specifically down-regulated in rice, whilst this was not seen for Arabidopsis (Figure 3). Of these, the opposite responses of heme (i.e. non-symbiotic hemoglobin) encoding genes was particularly noteworthy, as these are thought to have a role in NO scavenging and redox balance maintenance under low oxygen conditions in rice (Igamberdiev and Hill, 2009; Sturms et al., 2011). Specifically, the two genes encoding non-symbiotic hemoglobins were down-regulated by ~10-fold (LOC_Os03g12510.1) and ~90-fold (LOC_Os03g13140.1), whilst their Arabidopsis ortholog (At2g16060) was induced up to 34-fold under low oxygen conditions (Supplementary Tables 4, 6). In contrast, sphingolipid metabolism, aspartate and homoserine metabolism, as well as DUF26 receptor kinases were only enriched in the up-regulated gene-sets in rice (Figure 3). Whereas, genes encoding pectin esterases and wax related functions were only significantly over-represented in the down-regulated gene-sets from Arabidopsis, but this was not seen in rice (Figure 3). One of the most interesting observations was that the up-regulation of biotic stress-respiratory burst functions, heat shock factors, WRKY transcription factors and ARR transcription factors was also unique to the Arabidopsis response to hypoxia/anoxia (Figure 3). Given that these are typically up-regulated under various stress conditions, finding these specifically induced in Arabidopsis under low oxygen conditions suggests that the perception of low oxygen stress in Arabidopsis may overlap with its perception of other abiotic/biotic stresses.

### How does the low oxygen response compare to the abiotic stress responses?

**Figure 4:**
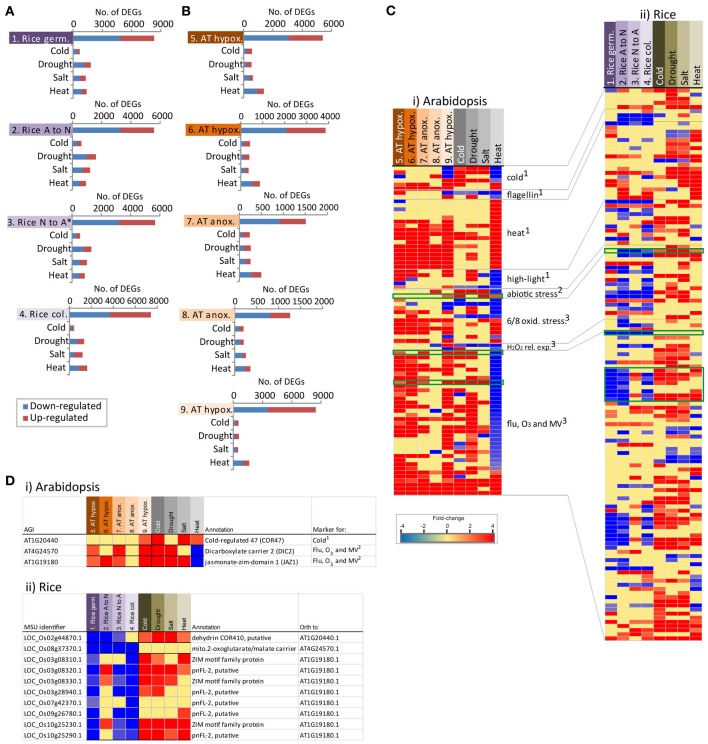
**Overlapping responses between low oxygen and abiotic stress in rice and Arabidopsis. (A)** Differentially expressed genes from each of the four low oxygen transcriptome datasets in rice (Lasanthi-Kudahettige et al., 2007; Narsai et al., 2009) were overlapped with the differentially expressed genes in response to cold, drought, salt, and heat stress (Jain et al., 2007; Hu et al., 2009). The numbers of genes showing overlapping responses are shown. The ^*^ indicates that the fold-changes were inversed for this comparison to show the response to anoxia. **(B)** Differentially expressed genes from each of the five low oxygen transcriptome datasets in Arabidopsis (Loreti et al., 2005; Branco-Price et al., 2008; Christianson et al., 2009, 2010; Banti et al., 2010) were overlapped with the differentially expressed genes in response to cold, drought, salt, and heat stress (Kilian et al., 2007). The number of genes showing overlapping responses is shown. (**Ci**) A heatmap showing differential expression of the 80 genes that were both significantly responsive to heat in Arabidopsis, as well as being previously identified as abiotic stress marker genes [by Gadjev et al. (2006), Lu et al. (2007), and Rasmussen et al. (2013) in Arabidopsis]. Expression is shown in response to low oxygen and abiotic stress. (**Cii**) A heatmap showing the expression of the 135 rice orthologs to the 80 Arabidopsis abiotic stress responsive genes (from **Ci**). (**Di**) The expression of three Arabidopsis genes representing examples of common transcriptomic responses to low oxygen and one or more abiotic stress in Arabidopsis. (**Dii**) Expression of the rice orthologs to the three Arabidopsis genes, showing that these differ in the transcriptomic responses to low oxygen and one or more abiotic stress in rice.

Given that the response to low oxygen stress showed some similarity to abiotic stress in Arabidopsis, we overlapped the lists of differentially expressed genes in response to low oxygen stress with abiotic stress including drought, salt, cold and heat stress. All data was analyzed in the same manner using the abiotic stress datasets outlined in Table 1 (Jain et al., 2007; Kilian et al., 2007; Hu et al., 2009). For each of the four anoxia comparisons individually in rice, the numbers of genes overlapping were mostly similar with the highest overlap seen with drought stress for three out of the four studies (Figure 4A). In contrast, the transcriptomic response to heat showed the highest number of overlapping genes across each of the five individual hypoxia/anoxia comparisons in Arabidopsis (Figure 4B). Given that this overlap is observed with heat stress in Arabidopsis, and the known link between the heat and anoxia response in Arabidopsis, we pursued this further by extracting at the heat responsive genes in Arabidopsis and examining these more closely. In an effort to see whether low oxygen conditions is perceived as an abiotic stress in Arabidopsis, particularly for heat stress, we extracted the differentially expressed genes in response to heat that were also known stress markers (Gadjev et al., 2006; Lu et al., 2007; Rasmussen et al., 2013) and viewed the expression changes in parallel with the responses to anoxia/hypoxia as well as other abiotic stresses (cold, drought, salt; Figure 4Ci). Specifically, these included the genes identified as abiotic stress marker genes (Lu et al., 2007) including, cold, drought, salt, and heat specific stress marker genes (Rasmussen et al., 2013) as well as oxidative stress markers genes (Gadjev et al., 2006). In this way 80 Arabidopsis genes are shown that are responsive to both low oxygen as well as abiotic stress (Figure 4Ci). Using sequence similarity [Gramene; (Jaiswal et al., 2006)] and Inparanoid (Ostlund et al., 2010), the 135 rice orthologs to the 80 Arabidopsis genes were also analyzed and shown in the same way (Figure 4Cii).

As expected, many of the rice orthologs were also induced under abiotic stress in rice (Figure 4Cii). However, it is also apparent that while many of these stress marker genes are also induced under the hypoxia/anoxia comparisons in Arabidopsis, this is not seen for rice, where many are in fact down-regulated in response to hypoxia (Figure 4C). Three examples of genes showing different/opposite responses between Arabidopsis and rice are indicated in the green boxes (Figure 4Cii) and closer examination of these is shown in Figures 4Di,ii, respectively. One of these genes encodes the cold-responsive marker gene in Arabidopsis (cold-regulated 47—COR47; At1g20440) (Guo et al., 1992; Lu et al., 2007), which is orthologous to LOC_Os02g44870.1 in rice and shows significant induction in both species under cold and other abiotic stresses (Figure 4D). However, while this gene was induced 2.5-fold under hypoxia in Arabidopsis, its ortholog was down-regulated between 2.5 and 4-fold in response to anoxia in rice (Figure 4D). Similarly, an oxidative stress marker gene in Arabidopsis (At4g24570) encoding a dicarboxylate carrier was induced under hypoxia (2.6-fold), anoxia (3.7-fold), cold (454-fold) and drought stress (40-fold) in Arabidopsis while its rice ortholog (LOC_Os08g37370.1) was unchanging under abiotic stress and down-regulated up to 12-fold under anoxia (Figure 4D). This was particularly interesting as this gene, among others (Figure 4Ci), was down-regulated 15-fold under heat-stress in Arabidopsis (Figure 4Di). Similarly another gene that was down-regulated 6-fold under heat stress, but up-regulated after hypoxia/anoxia (up to 6-fold), cold (31-fold), drought (41-fold), and salt (5-fold) stress in Arabidopsis was the jasmonate zim-domain containing gene (At1g19180) (Figure 4Di). Interestingly, the Gramene database (Jaiswal et al., 2006) shows eight zim-domain containing rice genes as orthologs to this Arabidopsis gene (Figure 4Dii). While seven out of eight of these were induced under abiotic stress and three were briefly induced in response to switching to anoxia, all eight genes were down-regulated in two or more anoxia comparisons (Figure 4Dii).

It was particularly interesting to find that the rice orthologs to the Arabidopsis abiotic stress markers, which were down-regulated under heat stress in Arabidopsis, are in fact down-regulated under anoxia in rice (Figures 4C,D). Specifically, it was evidenced that oxidative stress marker genes (flu, O_3_, and MV) in particular were down-regulated under heat stress and up-regulated under hypoxia/anoxia in Arabidopsis (Figure 4Ci), whilst the rice orthologs to these genes are down-regulated under anoxia (Figure 4Cii). It has previously been shown that under heat stress, there is a significant number of oppositely responsive orthologous genes between rice and Arabidopsis (Narsai et al., 2010). Specifically, it was shown that under heat stress, genes encoding redox functions are up-regulated in rice and down-regulated in Arabidopsis (Narsai et al., 2010). Thus, it was particularly interesting to see these genes were up-regulated under low oxygen conditions in Arabidopsis, whilst their rice orthologs are down-regulated (Figure 4C).

Using these parallel datasets to analyse the low oxygen response and other abiotic stress responses, it was possible to isolate and identify low oxygen marker genes in rice and Arabidopsis. To do this, the significantly differentially expressed genes were filtered to identify genes showing the highest fold induction in response to low oxygen in rice (>50-fold) in two or more out of the four low oxygen response studies/comparisons, whilst not showing up-regulation under abiotic stress (Table 2). Similarly, the significantly differentially expressed genes in Arabidopsis were filtered to identify genes showing the highest fold induction in response to low oxygen (>50-fold) in three or more out of the five low oxygen response studies/comparisons (Table 2). In this way, highly responsive low oxygen markers were identified in both rice and Arabidopsis (Table 2). Notably, pyruvate decarboxylase was identified as a marker of low oxygen stress in Arabidopsis, showing between a 17- and 586-fold induction under all five low oxygen comparisons (At4g33070; Table 2). Similarly, in rice, pyruvate decarboxylase was also induced under low oxygen, however, this was to a lower extent (not meeting the criteria as a marker) (Supplementary Table 4). Additionally, it was not surprising that an alpha amylase encoding gene (LOC_Os09g28340.1) was identified to be a low oxygen marker in rice, as these have been shown to have a crucial function in the metabolic changes that occur under low oxygen conditions in rice (Loreti et al., 2003). Similarly, it was not surprising to see a glycosyl hydrolase (LOC_Os11g47560.1) as a low oxygen marker gene in rice, as these are also known to be responsive under low oxygen conditions in rice (Table 2). Interestingly, no Arabidopsis orthologs were detected for the aforementioned alpha-amylase and glycosyl hydrolase using the Inparanoid method (Ostlund et al., 2010) or based on sequence homology using Gramene (Jaiswal et al., 2006), suggesting a more specific role for these in rice.

**Table 2:**
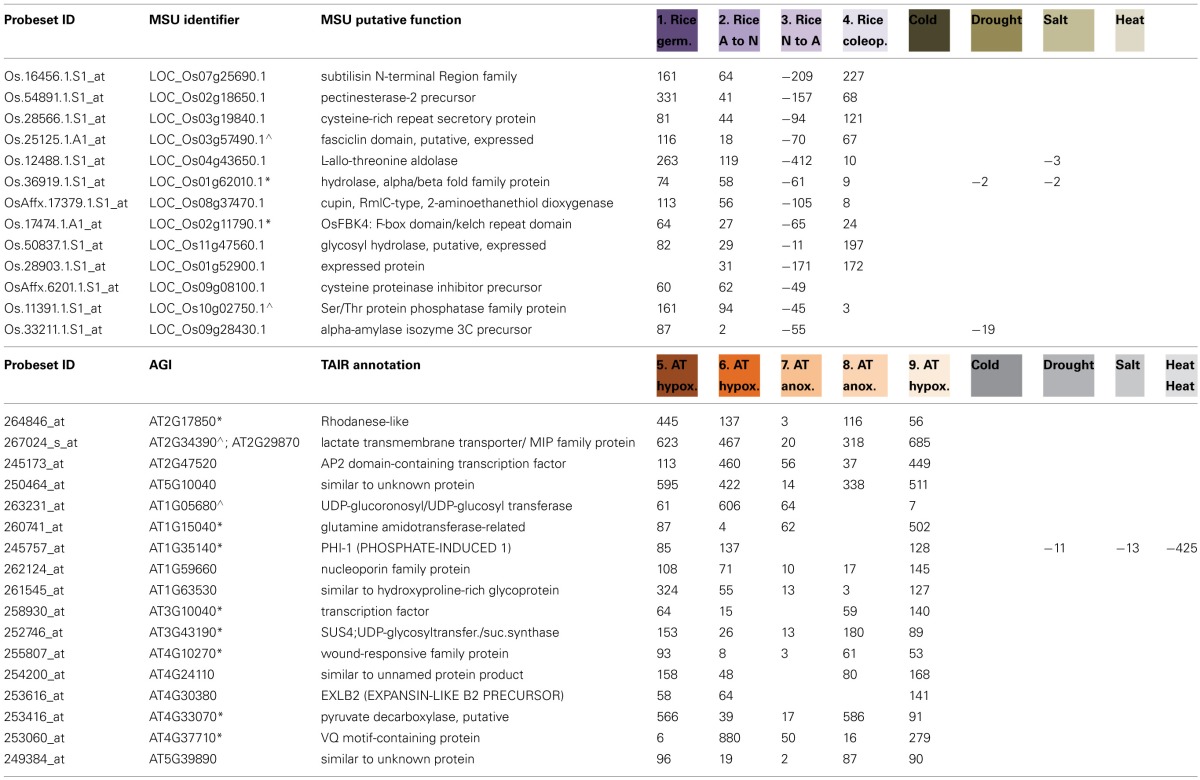
**Low oxygen marker genes in rice and Arabidopsis**.

For rice and Arabidopsis, various datasets examining the hypoxia/anoxia responses as well as abiotic stress responses were analyzed in parallel (details in Table 1). Top induced genes in response to low oxygen in rice and Arabidopsis were identified and the significant fold-changes after false discovery rate correction (p < 0.05, PPDE > 0.96) are shown. For rice, genes induced >50-fold in two or more of the rice anoxia studies are shown. For Arabidopsis, genes induced >50-fold in three or more hypoxia/anoxia studies are shown. These were also filtered exclude genes up-regulated under abiotic stress. An asterisk next to a gene indicates that the orthologous gene(s) from the other species (rice/Arabidopsis) is/are also up-regulated under low oxygen, whereas a ^∧^indicates that the orthologous gene is down-regulated.

Notably, the Arabidopsis gene At1g35140 encoding the phosphate induced (exordium-like EXL1) gene was also identified as a low oxygen marker (Table 2). This was particularly interesting given that Arabidopsis mutants of this gene have recently been shown to have reduced hypoxia tolerance (Schroder et al., 2011). Interestingly, it can be seen that while eight out of the 18 Arabidopsis low oxygen markers had rice orthologs that were also induced under low oxygen (indicated by an asterisk in Table 2), only two of the 12 low oxygen markers in rice had Arabidopsis orthologs showing conserved inductions under low oxygen (asterisk next to rice genes in Table 2). In addition, it was also seen that two rice genes had orthologs that were in fact down-regulated under low oxygen in Arabidopsis (Table 2). Namely; the fascilin domain containing gene LOC_Os03g57490.1 (induced between 18 and 116-fold; Table 2) is orthologous to At2g35860.1 (64% sequence similarity) which is down-regulated over 2.5 fold under low oxygen in two of the five Arabidopsis studies (Supplementary Table 6). Similarly, while the UDP-glucoronosyl transferase encoding gene At1g05680 was identified as a low oxygen induced marker in Arabidopsis (induced between 7 and 606-fold; Table 2), its rice ortholog LOC_Os04g12980.1 (41% sequence similarity) is down-regulated over 2.2-fold in response to anoxia in all four rice studies (Supplementary Table 4).

## Discussion

Germination is a crucial, energy demanding stage of the plant life cycle. Rice can not only survive under low oxygen conditions but can also germinate in the complete lack of oxygen (Howell et al., 2007; Bailey-Serres and Voesenek, 2008; Magneschi and Perata, 2008). Despite significant differences in the life cycle lengths or storage reserves (e.g., oil seed vs. starch seed) of different plant species, the process of germination often occurs rapidly. When germination under aerobic conditions in Arabidopsis (Narsai et al., 2011a), aerobic conditions in rice (Howell et al., 2009) and anaerobic conditions in rice were compared (Narsai et al., 2011a), core functions was altered in the transcriptomic responses, revealing conserved down-regulation of ABA responsive proteins, seed storage proteins and protein degradation, as well as the up-regulation of protein synthesis, lipid metabolism and cell wall functions (Figure 1). Thus, even under anaerobic conditions in rice, we found that there are several conserved transcriptomic responses that are likely to be crucial for germination progression in plants.

During anaerobic germination, in addition to the morphological adaptations including coleoptile elongation, it is known that carbohydrate metabolism is altered and fermentation is activated (Magneschi and Perata, 2008). As expected, closer examination under anaerobic germination also revealed an early induction of carbohydrate metabolism and glycolysis functions specific to anaerobic germination (Figure 2). Notably, in some cases, even when the induction of specific genes was seen between both species, the magnitude differed significantly. For example, while pyruvate decarboxylase was identified as a marker of low oxygen stress in Arabidopsis, showing a greater than 50-fold induction (Table 2), the induction of pyruvate decarboxylase in rice was much smaller. In contrast, pyruvate dikinase was also induced under low oxygen conditions in both species; however, this induction was much larger in rice compared to Arabidopsis. This was not entirely surprising, given that under low oxygen conditions in rice, it has been proposed that pyrophosphate may be used as an alternative energy currency over ATP, acting as a high-energy donor molecule (Huang et al., 2008; Igamberdiev and Kleczkowski, 2011). Hence, this alteration may help to maintain the energy balance, and tolerance of rice under low oxygen conditions.

Interestingly, a number of genes encoding PPR domain containing proteins were also found to be specifically induced under anaerobic germination in rice. In the past few years, the roles of PPR proteins have been better elucidated revealing functions in organelle RNA processing and editing (Saha et al., 2007). Interestingly, during Arabidopsis germination (i.e., under aerobic conditions) it was revealed that several PPR encoding genes show germination specific expression (Narsai et al., 2011a) and many of these are embryo lethal when a loss-of-function occurs (Tzafrir et al., 2003). Thus, the specific induction of these PPRs during anaerobic germination may be indicative of a crucial role for these PPRs during anaerobic germination in rice as well.

In addition, a specific suppression of genes encoding redox related functions were identified during anaerobic germination in rice, where these genes were not only down-regulated compared to aerobic germination but also down-regulated over the anaerobic germination time-course. This is particularly interesting given that cross-talk between NO and reactive oxygen species signaling has been shown to have a role in the light- and hormone-specific regulation of seed development and germination in plants [reviewed in Sirova et al. (2011)]. It has also been proposed that plant hemoglobins may modulate the effects of hormones that use NO as a signal transduction component (Hebelstrup et al., 2007). Thus, the specific suppression of redox functions, including hemoglobins in rice, may be part of the transcriptomic response to these signals coordinating anaerobic germination. Additionally, recent studies in rice are providing evidence supporting a crucial role of hemoglobins in NO scavenging, even reporting increased activity of these proteins in rice compared to their human counterparts under low oxygen conditions (Igamberdiev and Hill, 2009; Sturms et al., 2011). Furthermore, when the expression of hemoglobin genes were compared under low oxygen conditions in rice, Arabidopsis and poplar (which is also a flood tolerant species), it was seen that while the genes encoding non-symbiotic hemoglobins are suppressed in rice and poplar, they are significantly induced in Arabidopsis (Narsai et al., 2011b). This implies a controlled response to low oxygen, whereby suppressing hemoglobin gene expression may be more characteristic of low oxygen tolerant species.

Recent studies have shown a link between redox functions, anaerobiosis and heat stress in Arabidopsis (Pucciariello et al., 2012a). In 2008, it was shown that pre-treating Arabidopsis plants with heat stress resulted in the induction of heat shock factors and enabled greater hypoxia tolerance (Banti et al., 2008). Comparative analysis of the transcriptomic response to heat and anoxia/hypoxia in this study also confirmed this greater overlap with heat stress, compared to salt, drought and cold stress. In addition, a more Arabidopsis specific induction of heat shock factors was seen under the five Arabidopsis low oxygen studies, compared to rice (Figure 3). Confirmation for the role of heat shock factors in the low oxygen response also came when it was shown that HsfA2 enhances low oxygen tolerance by altering the expression of its target genes in Arabidopsis (Banti et al., 2010). Similarly, although the SUB1A locus was first described for its role in anoxia tolerance in rice (Xu et al., 2006), a more recent study has also revealed a role in drought tolerance (Fukao et al., 2011). Thus, there is cross-talk between the low oxygen and other abiotic stress responses.

Interestingly, when the expression of known heat-responsive abiotic stress marker genes in Arabidopsis (Gadjev et al., 2006; Lu et al., 2007; Rasmussen et al., 2013) were examined under low oxygen conditions in this study, strong induction of these was also seen under low oxygen conditions in Arabidopsis, whilst this was not seen for their rice orthologs. Specifically, it was seen that oxidative stress marker genes [flu, O_3_, and MV—identified in Gadjev et al. (2006)] were down-regulated under heat stress and up-regulated under low oxygen in Arabidopsis, whilst the rice orthologs to these genes were down-regulated under anoxia. These divergent expression responses for oxidative stress marker genes, suggests differences in oxidative/ROS signaling under low oxygen conditions between Arabidopsis and rice. In Arabidopsis, it has been shown that ROS are produced under heat stress and low oxygen conditions, and a mechanism linking ROS signaling with ERFs has been also been shown (Pucciariello et al., 2012a,b).

Since the identification of the role of the SUB1A ERF in anoxia tolerance in rice (Xu et al., 2006), a link between anoxia and ERFs has been founded. Recently, a role for group VII ERFs (containing a conserved N-terminal motif) in oxygen sensing and mediating the low oxygen response was shown in Arabidopsis (Gibbs et al., 2011). It was determined that under low oxygen conditions in Arabidopsis, the N-end rule pathway of targeted proteolysis acts as an oxygen sensor, where Arabidopsis plants lacking the constituents of this pathway were more hypoxia tolerant (Licausi et al., 2011). Notably, in comparison to rice, genes encoding ethylene signaling functions were more specifically induced in Arabidopsis, supporting the role for ethylene signaling in the Arabidopsis hypoxia response.

### Conclusions

In this study, core transcriptomic responses during germination in rice and Arabidopsis were identified. Comparison with other abiotic stress revealed some overlap with the low oxygen stress response, particularly for heat stress in Arabidopsis. Also, sets of low oxygen responsive markers were identified, both for Arabidopsis and rice, with two of the identified Arabidopsis markers (pyruvate decarboxylase—At4g33070 and phosphate induced EXL1—At1g35140) already known to function in the hypoxia response in Arabidopsis (Kursteiner et al., 2003; Schroder et al., 2011). The induction of HSFs and WRKY TFs were seen under heat and low oxygen stress in Arabidopsis, supporting the demonstrated role of these HSFs in heat and anoxia stress (Banti et al., 2008, 2010). While HSF functions were also first characterized for their role under other abiotic stresses, the crucial role of WRKY TFs under abiotic stress is also well-known (Chen et al., 2012). Thus, it is also worthwhile considering a potential role for these TFs in hypoxia tolerance in Arabidopsis as well. Overall, we have shown the different responses to low oxygen stress in rice and Arabidopsis and demonstrated the core transcriptional reprogramming that occurs as part of the hypoxia response in plants, including divergent responses between Arabidopsis and rice. Specifically, these findings revealed an interesting link between ROS and the anoxia response.

### A role for ROS in the anoxia response

While many plants, and indeed organisms, display common responses to low or no oxygen conditions, these common responses alone are not sufficient to explain anaerobic tolerance. A comparison of the unique responses in rice over germination with Arabidopsis, reveal that the suppression of a variety of genes associated with redox balance is unique to rice. Furthermore, heat treatment in Arabidopsis, also results in a suppression of genes associated with redox functions, and heat has been shown to increase tolerance to anaerobic conditions (Banti et al., 2008, 2010). In recent years it has been shown that ROS play essential roles not only in stress responses, but in also maintaining growth (Foreman et al., 2003). A ROS gradient from root tips defines proliferation and differentiation (expansion) (Tsukagoshi et al., 2010; Wells et al., 2010). To date this role is largely defined in roots, but the role of ROS, and different ROS species defining proliferation and expansion position them as key mediators between environmental stress signaling and growth promoting pathways. ROS have also been implicated in development in animals from Dictyostelium to mammals (Aguirre and Lambeth, 2010). From the analysis carried out above it is proposed that low oxygen conditions is not sensed as a stress in rice, in comparison to Arabidopsis where it is perceived as a stress. In Arabidopsis, this leads to an induction of stress and anti-oxidant defence systems, resulting in a cessation of growth. In rice, the suppression of transcripts for anti-oxidant defence systems will mean that ROS (or RNS) can still exist and act as an essential signal to drive the morphological changes that occur in rice under low oxygen conditions, thus, enabling growth to continue.

## Materials and methods

### Publically available microarray datasets

Publically available microarrays were downloaded from the Gene Expression Omnibus or MIAME Array Express Databases (for each species) and these were normalized together. Affymetrix Expression Console software was used to first obtain present, absent, marginal calls for gene expression following MAS5.0 normalization. Genes that were called present (*p* < 0.05) were kept for further analysis. Data was GC-RMA normalized using Partek Genomics Suite and this was used for the differential expression analysis. The microarrays used for the germination analyses included an aerobic germination time course in rice—E-MEXP-1766 (Howell et al., 2009), anaerobic germination in rice—E-MEXP-2267 (Narsai et al., 2009) and aerobic germination time course in Arabidopsis—GSE30223 (Narsai et al., 2011a). For the hypoxia/anoxia datasets only the control (air) v hypoxia/anoxia microarrays were analyzed for both species (details, references and accession are shown in Table 1). For the differential expression analysis in response to abiotic stress in rice, these appear as shown in (Narsai et al., 2010), which compared the responses to drought, salt, cold and heat stress in Arabidopsis and rice. Details are also shown in Table 1.

### Differential expression analysis

After all the arrays were pre-processed and normalized, differential expression analysis was carried out using the Cyber-T method (Baldi and Long, 2001; Long et al., 2001), as done in previous studies (Narsai et al., 2010, 2011a). Using the Cyber-T software for differential expression analysis, a gene was identified as significantly differentially expressed when *p* < 0.05 and the false discovery rate is less than 5% (PPDE > 0.96). For all the analyses shown in this study, this was further filtered to only include genes that were significantly differentially expressed by greater than 2-fold.

### Pageman analysis

All the differentially expressed genes were used for Pageman over-representation analysis (Usadel et al., 2006) as done in previous studies (Narsai et al., 2011a). ORA Fisher's test 2.0 was used to determine significant over-representation which calculates a *z*-score showing the over/under-representation of specific functional categories. *Z*-scores greater than 1.96 indicate significant over-representation at *p* < 0.05.

### Orthology between rice and arabidopsis genes

In order to identify rice orthologs to the Arabidopsis genes, two methods were employed based on; (1) sequence identity [extracted from Gramenemart; Jaiswal et al. (2006)] and (2) the Inparanoid method (Ostlund et al., 2010). If one or both of these methods identified a gene as orthologous to the Arabidopsis gene, these were considered rice orthologs.

### Conflict of interest statement

The authors declare that the research was conducted in the absence of any commercial or financial relationships that could be construed as a potential conflict of interest.

## Supplementary material

Supplementary Figure S1 **Pageman analysis of germination in rice (aerobic germination; Howell et al., 2009, anaerobic germination; Narsai et al., 2009) and Arabidopsis (Narsai et al., 2011a) using step-wise comparisons**. Over-representation analysis was carried out for genes significantly differentially expressed (>2-fold, *p* < 0.05, PPDE > 0.96) over the course of germination. Common over-represented functional categories across the three germination studies are shown. The *z*-scores indicating over/under representation of genes in each functional category are indicated as a heatmap where red indicates over-representation and blue indicates under-representation.

Supplementary Table 1 **The step-wise comparisons used for rice under aerobic (Howell et al., 2009) and anaerobic (Narsai et al., 2009) germination**. All microarrays for both species were GC-RMA normalized and analyzed in the same manner for differential expression using the Cyber-T method (*p* < 0.05, PPDE > 0.96).

Supplementary Table 2 **The step-wise comparisons used for Arabidopsis germination (Narsai et al., 2011a)**. All microarrays for both species were GC-RMA normalized and analyzed in the same manner for differential expression using the Cyber-T method (*p* < 0.05, PPDE > 0.96).

Supplementary Table 3 **Pageman analysis of rice aerobic and anaerobic germination was carried out together and then matched with the Pageman analysis of Arabidopsis germination to show represented functional categories over the course of germination**. All microarrays for both species were normalized and analyzed in the same manner for differential expression (i.e., FC = 2-fold, *p* < 0.05, PPDE > 0.96) and the same parameters were used for the Pageman analysis (i.e., ORA Fisher using 2.0 as a cut-off).

Supplementary Table 4 **The low oxygen and abiotic stress comparisons and used for rice from the sources outlined in Table 1**. All microarrays for both species were GC-RMA normalized and analyzed in the same manner for differential expression using the Cyber-T method (*p* < 0.05, PPDE > 0.96).

Supplementary Table 5 **Pageman analysis (ORA Fisher using 2.0 as a cut-off) of rice aerobic vs. anaerobic germination, as well as step-wise germination was carried out to show represented functional categories unique to anaerobic germination**. All microarrays were normalized and analyzed in the same manner for differential expression (i.e., FC = 2-fold, *p* < 0.05, PPDE > 0.96). Genes from these are shown in Figure 2.

Supplementary Table 6 **The low oxygen and abiotic stress comparisons and used for Arabidopsis from the sources outlined in Table 1**. All microarrays for both species were GC-RMA normalized and analyzed in the same manner for differential expression using the Cyber-T method (*p* < 0.05, PPDE > 0.96).

## Abbreviations

ABA: abscisic acid
AGI: Arabidopsis gene identifier
CAREs: *cis*-acting regulatory element(s)
CHO: carbohydrate
DEG: differentially expressed genes
ERF: ethylene response factor
FA: fatty acid
FDR: false discovery rate
GC-RMA: GC content based Robust Multi-array Average
GI: gene identifier
HSF: heat shock factor
MSU: Michigan State University (rice database)
PPDE: Posterior Probability of Differential Expression
PPR: pentatricopeptide repeat
ROS: reactive oxygen species
TAIR: The Arabidopsis Information Resource
TF: transcription factor.
