# Supplementary figures and images for: How unique is the low oxygen response? An analysis of the anaerobic response during germination and comparison with abiotic stress in rice and Arabidopsis
Source: Front Plant Sci. 2013 Oct 1;4:349. doi: 10.3389/fpls.2013.00349 (PMC3787303; doi:10.3389/fpls.2013.00349)

Supplementary Figure 1.

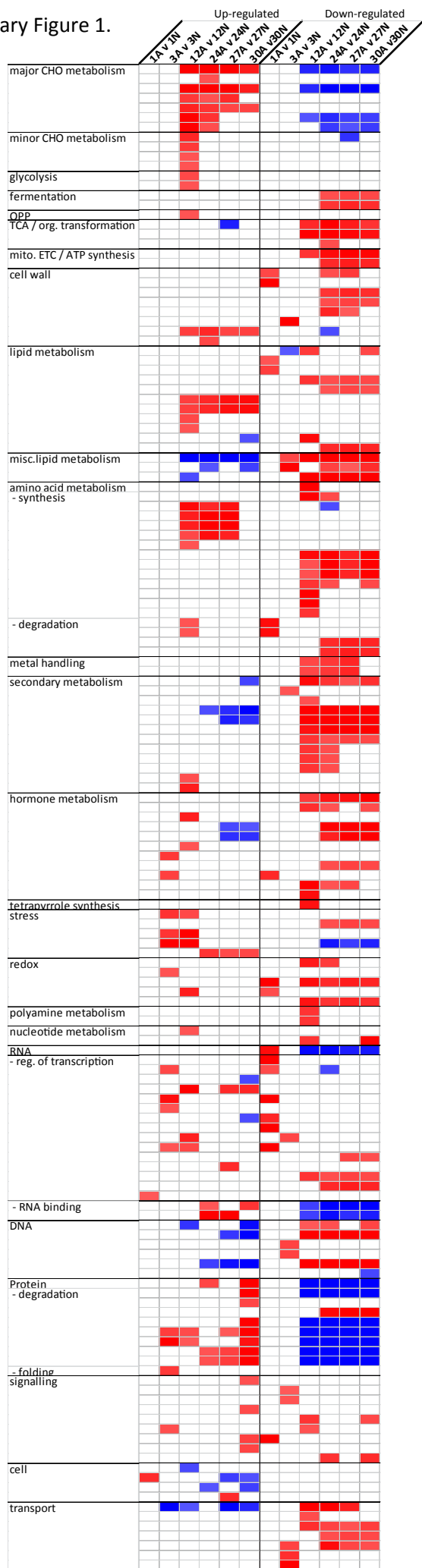

Supplement: Supplementary Figure S1 — Pageman analysis of germination in rice (aerobic germination; Howell et al., 2009, anaerobic germination; Narsai et al., 2009) and Arabidopsis (Narsai et al., 2011a) using step-wise comparisons. Over-representation analysis was carried out for genes significantly differentially expressed (>2-fold, p < 0.05, PPDE > 0.96) over the course of germination. Common over-represented functional categories across the three germination studies are shown. The z-scores indicating over/under representation of genes in each functional category are indicated as a heatmap where red indicates over-representation and blue indicates under-representation. [file Presentation1.PDF]
